# Supplementary material for: Within-Modality Synthesis and Novel Radiomic Evaluation of Brain MRI Scans
Source: Cancers (Basel). 2023 Jul 10;15(14):3565. doi: 10.3390/cancers15143565 (PMC10377568; doi:10.3390/cancers15143565)
Supplement: Supplementary file 1 [file cancers-15-03565-s001.zip › cancers-2434921-supplementary.pdf]

Within-Modality Synthesis and Novel Radiomic Evaluation of Brain MRI Scans

Supplementary S1

The radiomics features extracted in our study encompassed a total of twenty-three features, which were categorized into three groups: Gray Level Co-occurrence Matrix (GLCM), Gray Level Size Zone (GLSZM), and Gray Level Run Length Matrix (GLRLM). The GLCM features included Auto Correlation, Cluster Prominence, Cluster Shade, Contrast, Correlation, Energy, Entropy, and Homogeneity. The GLSZM features comprised Grey Level Mean, Grey Level Nonuniformity, Grey Level Variance, Large Zone Emphasis, Large Zone High Grey Level Emphasis, Large Zone Low Grey Level Emphasis, Low Grey Level Emphasis, and Grey Level Nonuniformity. Lastly, the GLRLM features consisted of High Grey Level Run Emphasis, Long Run High Grey Level Emphasis, Long Run Low Grey Level Emphasis, Low Gray Level Run Emphasis, Run Length Nonuniformity, and Short Run Emphasis.

GLCM (Gray Level Co-occurrence Matrix) Features:

1. Auto Correlation: Measures the similarity between the intensity values of adjacent voxels, indicating the spatial correlation of similar gray-level values.

2. Cluster Prominence: Reflects the skewness of the GLCM, indicating the presence of clusters with similar gray-level values.

3. Cluster Shade: Measures the skewness and asymmetry of the GLCM, providing information about the presence of clusters with different gray-level values.

4. Contrast: Quantifies the local variations in intensity between neighboring voxels.

5. Correlation: Describes the linear dependency between gray-level values of neighboring voxels, indicating the spatial correlation.

6. Energy: Represents the sum of squared elements in the GLCM, providing a measure of the image's homogeneity and texture uniformity.

7. Entropy: Measures the randomness and complexity of the texture, indicating the amount of information present in the image.

8. Homogeneity: Reflects the similarity of neighboring voxels in terms of their gray-level values.

GLSZM (Gray Level Size Zone) Features:

1. Grey Level Mean: Represents the average gray-level value within distinct zones of the image.

2. Grey Level Nonuniformity: Quantifies the variability of gray-level values within distinct zones.

3. Grey Level Variance: Measures the spread of gray-level values within distinct zones, indicating the heterogeneity of the texture.

4. Large Zone Emphasis: Reflects the presence of large zones with similar gray-level values.

5. Large Zone High Grey Level Emphasis: Represents the presence of large zones with high-intensity gray-level values.

6. Large Zone Low Grey Level Emphasis: Indicates the presence of large zones with low-intensity gray-level values.

7. Low Grey Level Emphasis: Measures the presence of zones with low-intensity gray-level values.

8. Grey Level Run Length Nonuniformity: Quantifies the variability in the size of zones with similar gray-level values.

GLRLM (Gray Level Run Length Matrix) Features:

1. High Grey Level Run Emphasis: Represents the presence of long homogeneous regions with high-intensity gray-level values.

2. Long Run High Grey Level Emphasis: Indicates the presence of long homogeneous regions with both high-intensity gray-level values and long runs.

3. Long Run Low Grey Level Emphasis: Reflects the presence of long homogeneous regions with both low-intensity gray-level values and long runs.

4. Low Gray Level Run Emphasis: Measures the presence of long homogeneous regions with low-intensity gray-level values.

5. Run Length Nonuniformity: Quantifies the variability in the lengths of homogeneous regions.

6. Short Run Emphasis: Represents the presence of short homogeneous regions, indicating the fine details in the texture.

These radiomics features provide valuable information about the texture, spatial relationships, and distribution of gray-level values within medical images. By extracting and analyzing these

features, researchers can gain insights into the underlying characteristics and heterogeneity of the imaged tissue or structures.

Supplementary S2

(a)

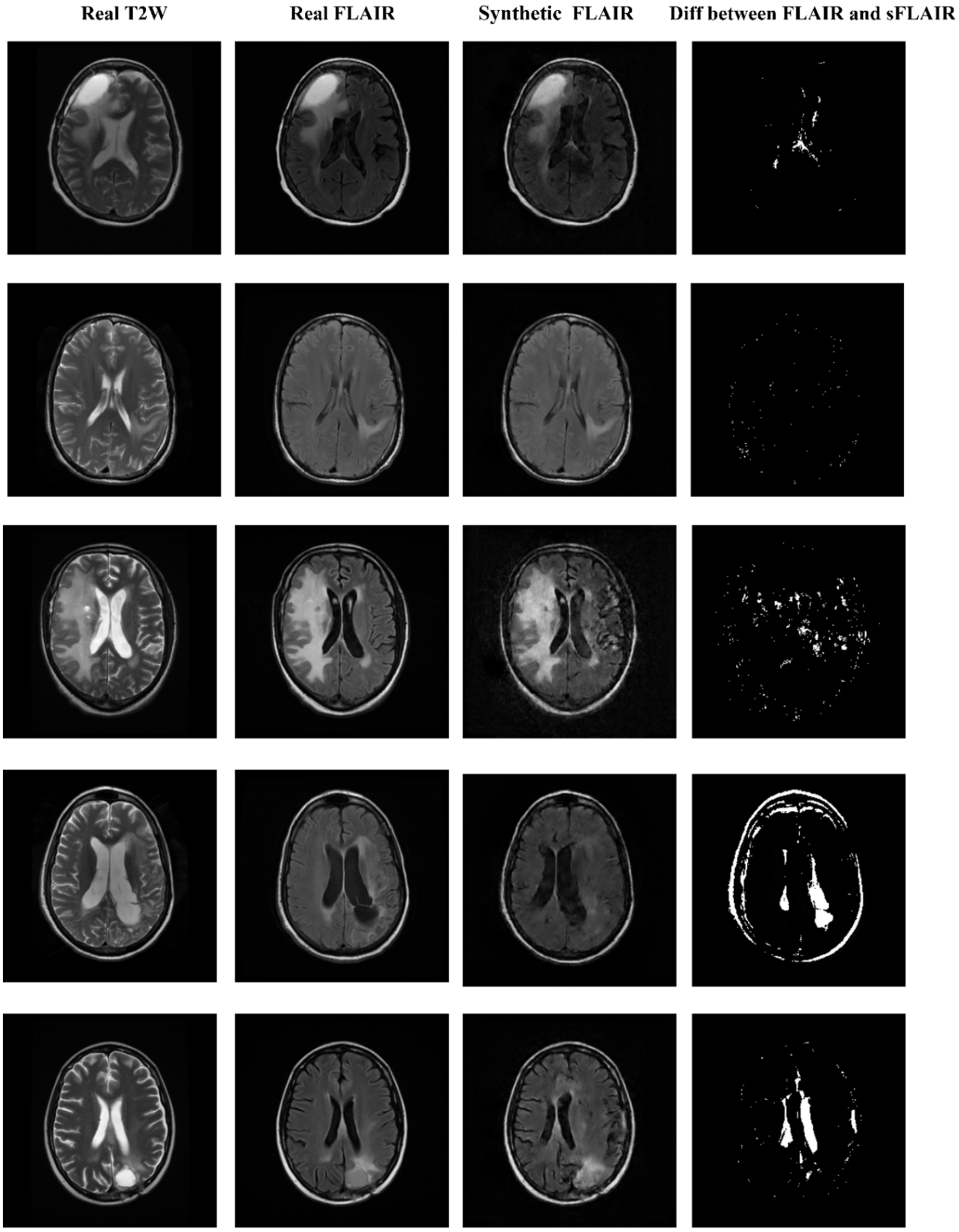

(b)

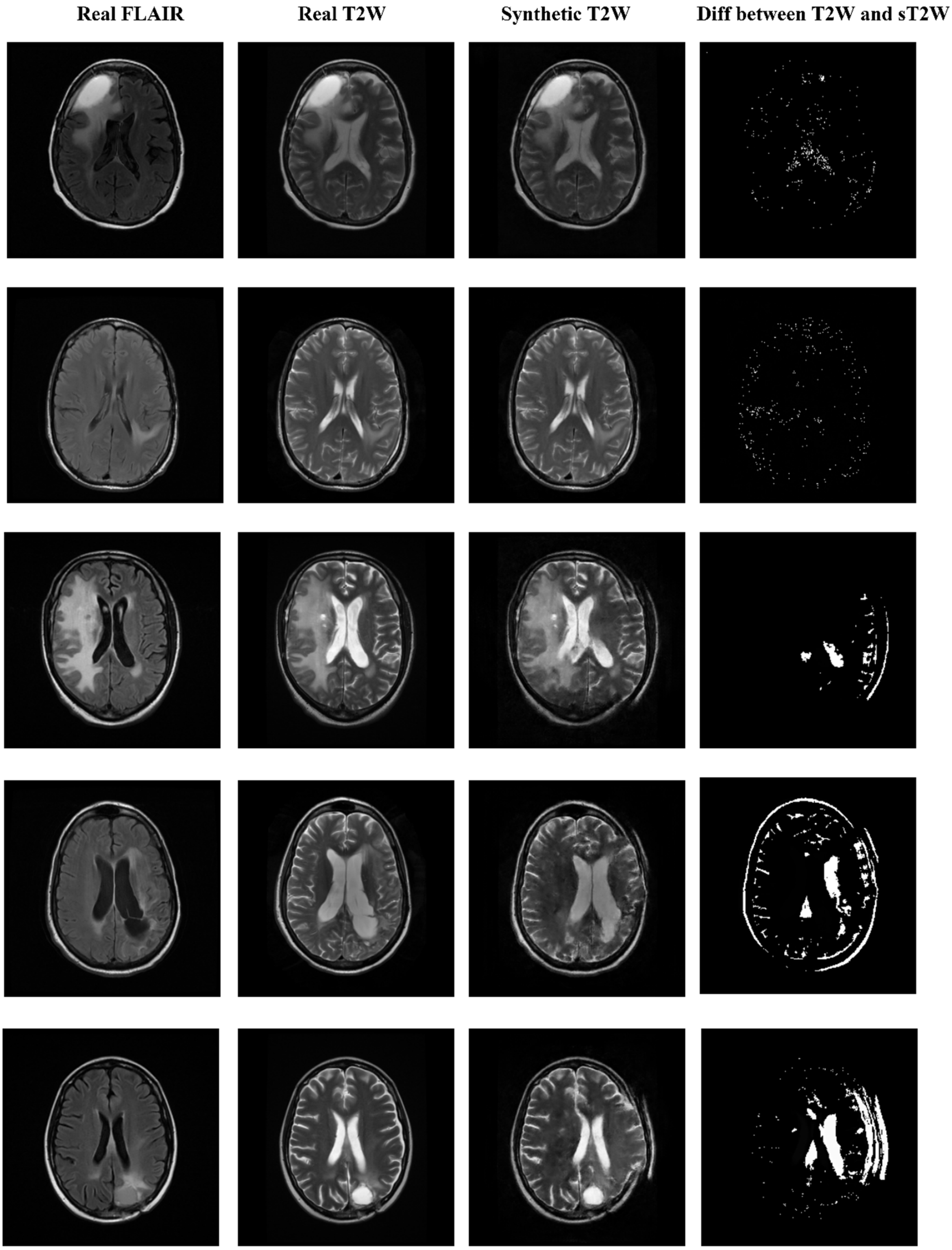

Figure S1. Qualitative comparison of CycleGAN model. There are five examples of T2W and FLAIR synthesis based on two mappings: Generating FLAIR Images from T2W Images (a), and Generating T2W Images from FLAIR Images (b).

(a)

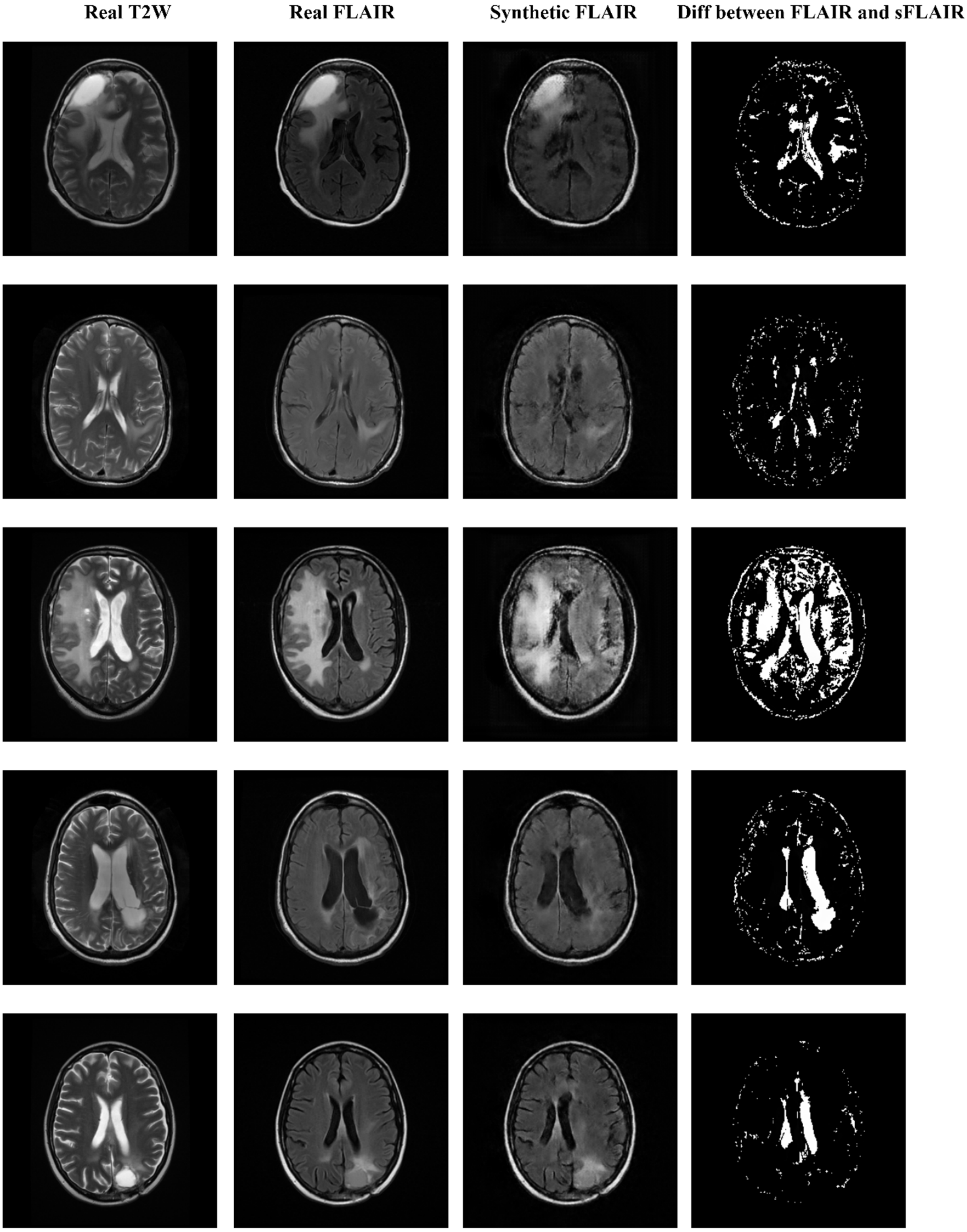
Real T2W
Real FLAIR
Synthetic FLAIR
Diff between FLAIR and sFLAIR

(b)

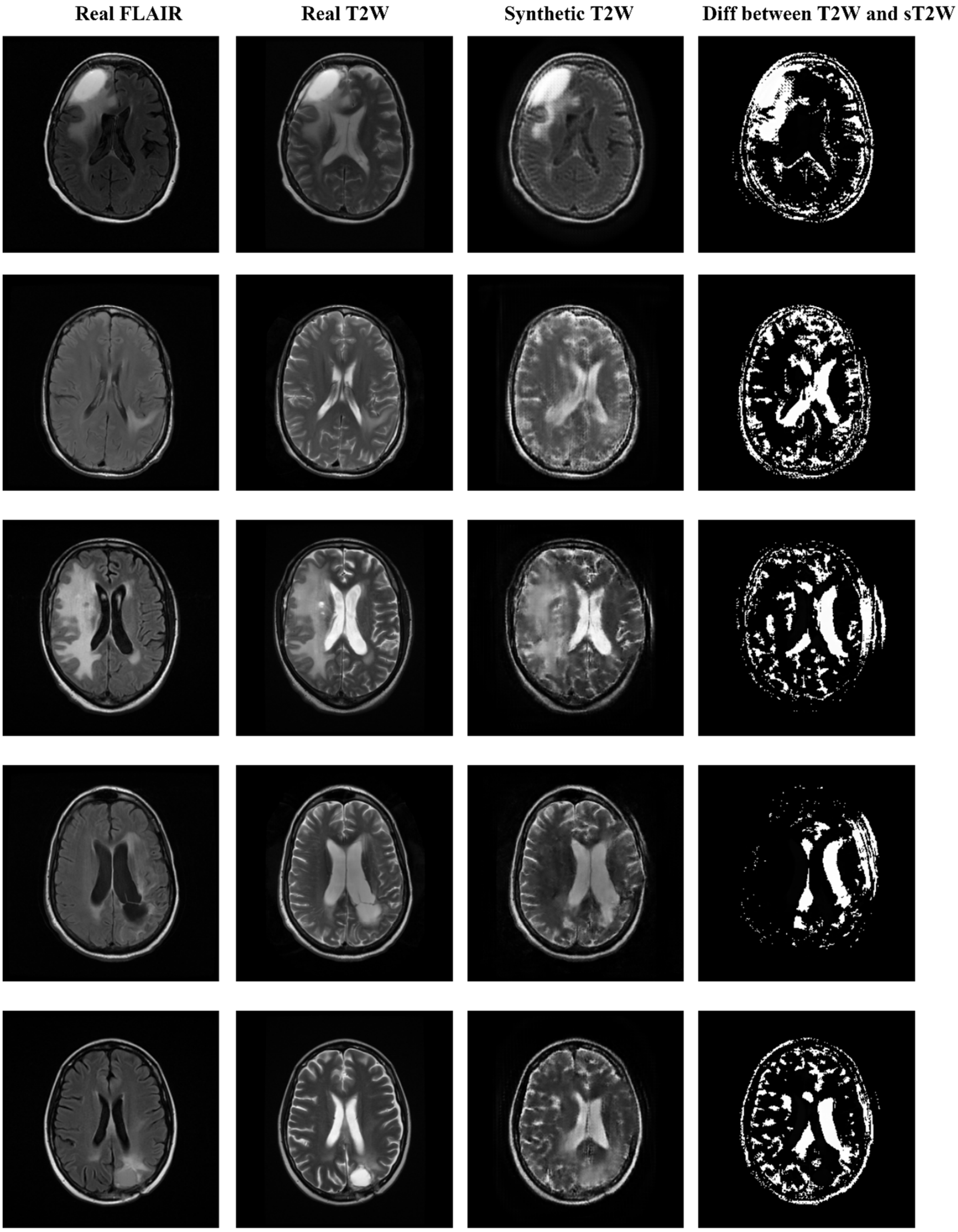

Figure S2. Qualitative comparison of the DC²Anet model. There are five examples of T2W and FLAIR synthesis based on two mappings: Generating FLAIR Images from T2W Images (a), and Generating T2W Images from FLAIR Images (b).
